# Anti-Inflammatory and Antioxidant Effects of Quercetin, Luteolin, and Proanthocyanidins in Canine PBMCs Stimulated with *Escherichia coli*

**DOI:** 10.3390/ani15243622

**Published:** 2025-12-16

**Authors:** Alma Virág Móritz, Viktória Fónagy, Roland Psáder, Ákos Jerzsele, Orsolya Farkas

**Affiliations:** 1 Department of Pharmacology and Toxicology, University of Veterinary Medicine, 1078 Budapest, Hungary; fonagy.viktoria@student.univet.hu (V.F.); jerzsele.akos@univet.hu (Á.J.);; 2 National Laboratory of Infectious Animal Diseases, Antimicrobial Resistance, Veterinary Public Health and Food Chain Safety, University of Veterinary Medicine, 1078 Budapest, Hungary; 3 Department of Internal Medicine, University of Veterinary Medicine, 1078 Budapest, Hungary; psader.roland@univet.hu

**Keywords:** canine PBMCs, *Escherichia coli*, flavonoids, quercetin, luteolin, proanthocyanidins, oxidative stress, TNF-α

## Abstract

Chronic gastrointestinal disorders in dogs are complex conditions often associated with oxidative stress, dysbiosis, and persistent immune activation, which can impair intestinal barrier integrity and promote bacterial translocation. Among intestinal bacteria, *Escherichia coli* may act as an opportunistic pathogen under inflammatory conditions and contribute to systemic inflammation. This study aimed to establish an in vitro model using primary canine peripheral blood mononuclear cells stimulated with canine-derived *E. coli* strains to investigate inflammatory and oxidative responses, and to evaluate the anti-inflammatory and antioxidant effects of selected flavonoids. Inflammatory activation was strongest following exposure to the enrofloxacin-inhibited *E. coli* 863 strain for 24 h. Treatments with quercetin, luteolin, and grape seed proanthocyanidins at concentrations of 25 and 50 µg/mL were then assessed. Minimum inhibitory and mutant prevention concentrations, intracellular reactive oxygen species, and tumor necrosis factor-α levels were determined. Although the tested flavonoids showed moderate antibacterial activity, all significantly reduced intracellular oxidative stress and inflammatory cytokine production compared to infected controls. These findings suggest that dietary flavonoids may exert beneficial immunomodulatory effects and could serve as potential adjuncts in managing canine chronic gastrointestinal inflammation. Further in vitro and in vivo validation is required to confirm their therapeutic relevance.

## Simple Summary

Chronic gut problems are common in dogs and can develop when harmful bacteria, stress, and long-term inflammation damage the intestinal wall. This damage permits opportunistic bacteria, including *Escherichia coli*, to sustain inflammatory processes and, under certain conditions, may enable their translocation into the bloodstream. In this study, the anti-inflammatory and antioxidant effects of natural plant-derived flavonoids were evaluated. Canine immune cells were utilized to assess their response to *Escherichia coli* infection, and three flavonoids—quercetin, luteolin, and grape seed proanthocyanidins—were tested. The tested flavonoids displayed limited antibacterial activity, observable only at higher concentrations, while they significantly reduced inflammatory responses and oxidative stress in the cells. The results suggest that flavonoids could be useful as natural supplements to support the treatment of long-term gut diseases in dogs by lowering the effects of inflammation and oxidative stress. More research is needed to confirm these findings.

## 1. Introduction

Chronic inflammatory enteropathies (CIE) are among the most common gastrointestinal (GI) disorders in dogs, characterized by persistent inflammation of the small intestinal mucosa. The underlying mechanism involves an immune-mediated inflammatory response that compromises the intestinal barrier, disrupts nutrient absorption, and leads to mucosal damage. Over time, these chronic inflammatory processes can cause structural alterations, including crypt hyperplasia, mucosal atrophy, and lymphoplasmacytic infiltration [1]. The disease is often associated with dysbiosis [2], and a breed-related genetic predisposition has also been suggested, indicating that certain breeds are more prone to chronic enteropathies [3]. In most cases, etiology is multifactorial, involving the interplay of several pathogenic mechanisms, which complicate diagnosis and therapy [1]. Typical clinical manifestations include persistent or recurrent diarrhea, weight loss, reduced body condition, bloating, vomiting, and occasionally hypoalbuminemia [3,4]. Diagnostic workup typically includes biochemical profiling, fecal examination for parasites, ultrasonography, and radiography, but definitive diagnosis requires endoscopy and histopathology [5]. Treatment is often empirical due to the variable and complex etiology. Initial management usually involves a hypoallergenic diet; if dietary intervention fails, pharmacological therapy with anti-inflammatory or immunosuppressive agents is indicated to mitigate the aberrant immune response [3]. Current therapeutic approaches primarily aim to correct dysbiosis, with increasing emphasis on microbiome-modulating interventions such as fecal microbiota transplantation [6].

In a healthy canine gut, the predominant bacterial phyla include *Bacillota*, *Fusobacteria*, *Bacteroidetes*, *Proteobacteria*, and *Actinobacteria*. The intestinal microbiota plays a crucial role in maintaining host health by supporting nutrient absorption, energy transport, metabolic homeostasis, protection against pathogens, and immune system modulation [7]. Alongside beneficial commensals, facultative pathogenic bacteria such as *Escherichia coli* (*E. coli*) are also present [8]. Disruption of this delicate microbial balance can contribute to intestinal barrier dysfunction. Tight junctions between intestinal epithelial cells play a central role in regulating permeability, preventing the translocation of toxins, harmful molecules, and pathogens across the gut wall. Impaired barrier integrity, regardless of its origin, facilitates bacterial translocation and promotes inflammatory disorders [9]. Consequently, chronic inflammatory enteropathies are often accompanied by such barrier dysfunction, creating a self-perpetuating cycle of dysbiosis, immune activation, and mucosal injury.

Bacterial translocation from the intestinal lumen poses a significant risk in dogs with chronic enteropathies, as it can lead to systemic inflammation and secondary infections. Among the opportunistic pathogens involved, *E. coli* is one of the most frequently detected bacteria associated with intestinal dysbiosis and extra-intestinal infections [10]. *E. coli* is a Gram-negative, rod-shaped bacterium belonging to the Enterobacteriaceae family. It is a normal constituent of gut microbiota but may become pathogenic under dysbiotic conditions, causing a range of infections including gastrointestinal, urinary tract, respiratory infections, meningitis, and sepsis [11]. Among the enteric pathogens, enteropathogenic *E. coli* (EPEC) strains adhere to intestinal epithelial cells, leading to ultrastructural damage of the villi. Enterotoxigenic *E. coli* (ETEC) strains produce adhesins and heat-labile or heat-stable enterotoxins encoded by plasmids, which, despite not causing major histological damage, result in excessive fluid secretion and impaired nutrient absorption [12]. Although these specific pathotypes are classically associated with intestinal inflammation, *E. coli* does not necessarily need to belong to these pathogenic groups to exert detrimental effects on the immune system. Even non-pathogenic strains can contribute to inflammation when epithelial integrity is compromised, allowing bacterial components such as lipopolysaccharides (LPS) to translocate across the intestinal barrier.

The LPS is composed of lipid A, a core oligosaccharide, and a variable O-antigen. LPS acts as a biologically active endotoxin recognized by the LPS-binding protein in plasma, which, together with CD14 and MD2 cofactors, activates the TLR4 receptor. This recognition triggers intracellular signaling cascades such as NF-κB activation, resulting in the production of proinflammatory cytokines and chemokines that recruit leukocytes to the site of infection [13]. Clinically, LPS becomes relevant when intestinal barrier failure allows endotoxin translocation into systemic circulation. While local infections elicit a controlled immune response, systemic exposure leads to excessive inflammation, cytokine storm, increased vascular permeability, hypotension, and multiorgan dysfunction [14,15]. Severe barrier disruption may permit *E. coli* translocation into the bloodstream, triggering sepsis—a life-threatening condition characterized by an uncontrolled immune response and systemic inflammation. The ensuing release of cytokines and chemokines induces vasodilation, capillary leakage, microcirculatory failure, and tissue hypoxia, leading to cellular injury and organ failure. Coagulation system activation further exacerbates the condition through microthrombosis, endothelial injury, and disseminated intravascular coagulation [15]. The clinical signs of sepsis include fever, tachycardia, tachypnea, hypotension, and organ dysfunction. Dogs often develop systemic inflammatory response syndrome (SIRS) as a hyperinflammatory manifestation [12]. Hypoperfusion of vital organs leads to acute kidney injury, hepatic dysfunction, and the accumulation of metabolic toxins, while pulmonary involvement frequently results in acute respiratory distress syndrome. Early antimicrobial intervention was previously associated with improved outcomes [16]. However, due to the increasing prevalence of antibiotic-resistant *E. coli* strains and the limited recommendations for routine antibiotic use, alternative strategies to manage dysbiosis are gaining more attention. Antibiotic resistance arises through natural selection within bacterial populations [17], as resistant variants survive and proliferate following antimicrobial exposure [6]. Genetic mutations or horizontal gene transfer via plasmids or bacteriophages facilitate the spread of resistance even between distinct species [18,19]. Inappropriate antibiotic use, including unnecessary or insufficient treatments, improper dosage, or short therapy duration, accelerates the emergence of resistance [6]. Multidrug-resistant (MDR) strains, such as extended-spectrum β-lactamase (ESBL)-producing *E. coli*, are of particular concern [19]. According to the World Health Organization, antimicrobial resistance ranks among the top ten global public health threats [20,21]. In cases of severe *E. coli* septicemia, enrofloxacin is often used as a first-line treatment. Enrofloxacin, a fluoroquinolone antibiotic, exhibits broad-spectrum, concentration-dependent bactericidal activity [22]. Its mechanism of action involves inhibition of bacterial DNA topoisomerase II and IV, leading to DNA replication failure and bacterial death. Due to the rising fluoroquinolone resistance among *E. coli* isolates, its clinical use should be restricted to life-threatening infections or cases unresponsive to other empirical therapies [23].

Given the escalating problem of antibiotic resistance, there is growing interest in natural bioactive compounds, particularly flavonoids, as potential therapeutic adjuncts. Flavonoids are plant-derived secondary metabolites with notable antioxidant, immunomodulatory, and antimicrobial properties. They protect plant cells from oxidative stress, UV radiation, and microbial invasion by neutralizing reactive oxygen species (ROS) and maintaining cellular integrity [24]. Extensive studies have demonstrated that flavonoids can modulate intracellular signaling pathways, suppress proinflammatory cytokine production, and inhibit key enzymes such as cyclooxygenase and lipoxygenase, thereby alleviating chronic inflammation [25,26,27]. In addition to their antioxidant effects, flavonoids exhibit antimicrobial activity by inhibiting bacterial growth, biofilm formation, and modulate the gut microbiota composition [24]. Their bioavailability varies, and metabolic conjugation often alters their biological activity [28]. Quercetin, a flavonol-type polyphenol, possesses potent free radical scavenging capacity due to multiple hydroxyl groups. It has demonstrated protective effects in osteoporosis, cancer, and cardiovascular diseases [29]. By neutralizing ROS and enhancing antioxidant enzyme activity, quercetin helps maintain redox balance and protect cellular macromolecules from oxidative damage. Luteolin acts through both direct ROS scavenging and upregulation of antioxidant enzymes such as superoxide dismutase and glutathione peroxidase. It also inhibits NF-κB activation and modulates MAPK signaling, thereby helping reduce inflammatory cytokine production [30]. Mechanistically, quercetin has been reported to mitigate oxidative stress by activating the Nrf2 pathway and limiting oxidative injury programs such as ferroptosis, supporting its role as a regulator of cellular redox homeostasis [31]. Grape seed proanthocyanidins, belonging to the polyphenol family, are particularly effective antioxidants with stronger radical-scavenging capacity compared with many other flavonoids [24]. They inhibit lipid peroxidation, stabilize cellular membranes, enhance endogenous antioxidant defense systems, and beneficially modulate gut microbiota by reducing pathogenic bacteria and promoting beneficial species. This contributes to intestinal barrier maintenance and mitigates inflammation by stabilizing microbiome and educing endotoxin [32].

To model these systemic immune processes in vitro, primary canine peripheral blood mononuclear cells (PBMCs) were employed, as they comprise key components of both the innate and adaptive immune systems. PBMCs include lymphocytes (T, B, and NK cells), monocytes, and small numbers of dendritic cells, making them a relevant tool for evaluating the immunomodulatory potential of flavonoids under inflammatory conditions [33].

The adjunctive use of flavonoids represents a promising therapeutic approach, as their antioxidant and anti-inflammatory properties may enhance antibiotic efficacy and improve treatment outcomes. The aim of this study was to investigate the extent to which flavonoids can support host defense mechanisms. Using an in vitro model of bacterially induced oxidative and inflammatory stress, we examined the immunomodulatory and antibacterial potential of selected flavonoids. We hypothesized that, through their antioxidant and anti-inflammatory actions, flavonoids could strengthen the host defense system and potentially augment the effectiveness of conventional antibiotic therapy.

## 2. Materials and Methods

### 2.1. Isolation of PBMCs

Peripheral blood was collected from a healthy dog under regular veterinary supervision, with written owner consent obtained prior to sampling. The experimental protocol complied with national and international regulations and institutional ethical standards. The study was approved by the Food Chain Safety, Plant Protection and Soil Conservation Department of the Government Office of Pest County, Hungary (permit number: PE/EA/00980–6/2022).

Blood samples were collected in sterile EDTA-coated tubes. PBMCs were isolated using Histopaque 1077 (Merck, Darmstadt, Germany) according to the manufacturer’s protocol. Briefly, 3 mL of Histopaque 1077 was pipetted into a 15 mL centrifuge tube and overlaid with 3 mL of whole blood. The tubes were centrifuged at 400× *g* for 30 min at 23 °C. The mononuclear cell layer formed at the plasma–Histopaque interface was carefully aspirated and transferred to a new centrifuge tube. Cells were washed twice with 10 mL phosphate-buffered saline (PBS) and once with 5 mL PBS, centrifuged at 250× *g* for 10 min each time, and the supernatant was discarded. The final PBMC pellet was resuspended in RPMI-1640 medium (Merck, Darmstadt, Germany) containing L-glutamine and sodium bicarbonate, supplemented with 10% fetal bovine serum (FBS; EuroClone, Pero, Italy) and 1% penicillin–streptomycin solution (Lonza, Verviers, Belgium). The cell suspension was incubated overnight at 37 °C in a humidified atmosphere with 5% CO_2_. Cell number and viability were determined by trypan blue exclusion (Merck, Darmstadt, Germany) using a Bürker chamber, and the cell density was adjusted to 2 × 10^5^ cells/mL. Morphology was examined by DiffQuick staining (Siemens Healthineer, Erlangen, Germany). Cells were seeded in 24- and 96-well culture plates for subsequent assays.

### 2.2. E. coli and Flavonoid Treatments

Three canine-derived *E. coli* strains (isolates 340, 404, and 863) obtained from systemic infections were provided by the Department of Epidemiology and Microbiology, University of Veterinary Medicine Budapest. Bacteria were cultured from agar plates into Mueller–Hinton broth and incubated for 12 h, and then diluted in sterile PBS. For treatments with live bacteria, diluted bacterial suspensions were added to PBMC cultures at a 1:100 ratio and incubated for 1 or 5 h. In a second treatment group, bacterial growth was inhibited by adding enrofloxacin (0.5 µg/mL) to the PBMC–bacteria co-cultures, which were then incubated for 1, 5, or 24 h. In a third treatment, bacterial suspensions were heat-inactivated by autoclaving at 121 °C for 30 min, diluted 1:100, and added to PBMCs for 1, 5, or 24 h. Negative controls consisted of PBMCs cultured in medium only.

After optimizing experimental conditions, PBMCs were treated with selected flavonoids—quercetin (≥95%, Sigma-Aldrich, Darmstadt, Germany), luteolin (≥98%, Sigma-Aldrich, Darmstadt, Germany), and purified grape seed proanthocyanidins (≥98.8%, USP, Rockville, MD, USA)—at 25 and 50 µg/mL to assess their antioxidant and anti-inflammatory effects. Stock solutions were freshly prepared in 1% sterile dimethyl sulfoxide (DMSO; Sigma-Aldrich, Darmstadt, Germany) immediately before use [34,35].

### 2.3. Assessment of Cell Viability

The effects of the bacterial treatments and flavonoid treatments on cell viability were assessed by measuring LDH activity, using a commercial LDH Activity Assay Kit (Sigma-Aldrich, Hamburg, Germany) according to the manufacturer’s instructions. In this assay, the amount of lactate dehydrogenase released into the culture medium upon loss of plasma membrane integrity was quantified. Since LDH is a stable cytosolic enzyme that rapidly leaks from damaged or lysed cells, the level detected in the supernatant was taken to be proportional to the extent of cell death, allowing a sensitive and quantitative evaluation of cytotoxicity. For the assay, 10 μL of culture supernatant from each well was collected and added to the reaction mixture. The reaction mixture was prepared immediately before use according to the manufacturer’s protocol. The kit contained an LDH substrate solution (lactate), a cofactor mix (NAD^+^), and a colorimetric indicator dye/coupling reagent; these components were combined to form the working reagent that enables the LDH-catalyzed conversion of lactate to pyruvate, coupled with NAD^+^ reduction and subsequent formation of a colored formazan product. Then, 100 μL of the freshly prepared working reagent was dispensed into each well of a 96-well plate containing the 10 μL sample. The plate was incubated at 37 °C for 30 min, protected from light. After incubation, absorbance was measured at 450 nm using a microplate reader (SpectraMax iD3, Molecular Devices, San Jose, CA, USA). The degree of cell death was calculated based on LDH activity relative to the untreated control group and expressed as percentage cytotoxicity or fold change over control.

The effect of bacterial treatments on PBMC metabolic activity was evaluated using the Cell Counting Kit-8 (CCK-8; Dojindo, Munich, Germany), prior to ROS detection. The assay is based on the reduction of the water-soluble tetrazolium salt WST-8 by mitochondrial dehydrogenases to a formazan dye, the absorbance of which is proportional to cell viability and metabolic activity. The possible cytotoxic effect of enrofloxacin alone was also examined. Since the safety of quercetin, luteolin, and proanthocyanidins had been previously characterized in our laboratory, they were not re-evaluated in this study. The assay was performed according to the manufacturer’s instructions. Ten µL of CCK-8 reagent was added to each well of a 96-well plate and incubated for 1 h at 37 °C in 5% CO_2_. Absorbance was measured at 450 nm using a microplate reader (SpectraMax iD3, Molecular Devices, San Jose, CA, USA).

### 2.4. Measurements of Extracellular Hydrogen Peroxide and Intracellular ROS

Hydrogen peroxide levels in the culture supernatants were determined using the Amplex Red Hydrogen Peroxide/Peroxidase Assay Kit (Thermo Fisher Scientific, Waltham, MA, USA) following the manufacturer’s instructions. The assay is based on the HRP-catalyzed oxidation of the non-fluorescent Amplex Red reagent to the highly fluorescent product resorufin, allowing sensitive detection of H_2_O_2_. A 10 mM Amplex Red stock solution was freshly prepared in DMSO on the day of the experiment, and the working reagent was assembled by mixing the stock solution with 1× reaction buffer and horseradish peroxidase. For each well, 50–100 μL of culture supernatant was added to the reaction mixture in a black 96-well plate, followed by incubation for 30 min at 37 °C protected from light. Fluorescence was then measured at an excitation wavelength of 530–560 nm and emission at 590 nm using a microplate reader (SpectraMax iD3, Molecular Devices, San Jose, CA, USA). Hydrogen peroxide concentrations were calculated from a standard curve after subtraction of background fluorescence.

Intracellular reactive oxygen species (ROS) levels were quantified using 2′,7′-dichlorodihydrofluorescein diacetate (DCFH-DA) according to the manufacturer’s instructions. DCFH-DA is a non-fluorescent compound that diffuses through the cell membrane and is deacetylated by intracellular esterases to non-fluorescent DCFH. In the presence of ROS, DCFH is oxidized to fluorescent dichlorofluorescein (DCF), and the resulting fluorescence intensity is proportional to intracellular ROS generation. After treatments, cells were incubated with 10 µM DCFH-DA for 1 h at 37 °C in the dark. Following incubation, the dye-containing medium was removed, cells were washed with PBS, lysed with M-PER reagent, scraped, and the lysates were collected into microtubes and centrifuged at 1000× *g* for 10 min at 4 °C. The fluorescence intensity of the supernatants was measured using a microplate reader (SpectraMax iD3, Molecular Devices, San Jose, CA, USA) at 485 nm excitation and 535 nm emission.

### 2.5. Determination of TNF-α

Before ROS measurement, culture supernatants were collected, filtered through 0.22 µm sterile filters, and stored at −81 °C until analysis. Tumor necrosis factor-α (TNF-α) concentrations were quantified using a commercial canine sandwich enzyme-linked immunosorbent assay (ELISA; ElabScience Bionovation Inc., Houston, TX, USA) following the manufacturer’s instructions. Absorbance was read at 450 nm (SpectraMax iD3, Molecular Devices, San Jose, CA, USA). Calibration curves were used to convert absorbance values to concentrations (pg/mL), and the results were normalized to the untreated control values and expressed as percentages.

### 2.6. Microbiological Analyses of the Investigated Flavonoids

The minimum inhibitory concentration (MIC) and mutant prevention concentration (MPC) of quercetin, luteolin, and grape seed proanthocyanidins were determined against canine-derived *E. coli* strains using the broth microdilution method according to the Clinical and Laboratory Standards Institute (CLSI) guidelines. Bacterial cultures were adjusted to a turbidity equivalent to a 0.5 McFarland standard (≈1–2 × 10^8^ CFU/mL). For MIC testing, serial twofold dilutions of the test compounds and enrofloxacin were prepared in 96-well microtiter plates, and 10 μL of the 200-fold diluted bacterial suspension was added to each well. After 18 h of incubation, MIC values were determined by visual inspection. The MIC was defined as the lowest concentration that completely inhibited visible bacterial growth, corresponding to the first clear well adjacent to a turbid one.

For MPC determination, bacterial suspensions were adjusted to a 1 McFarland standard and inoculated into 96-well plates prepared as described for the MIC assay. Plates were incubated for 72 h, and bacterial growth was visually assessed.

To evaluate potential synergistic effects, the fractional inhibitory concentration (FIC) index was calculated to quantify the interactions between combined treatments. Enrofloxacin was tested in combination with each flavonoid (quercetin, luteolin, or proanthocyanidins) using serial twofold dilutions. The FIC of each compound was determined by dividing the MIC of the compound in combination by its MIC when tested alone. The sum of the individual FICs provided the overall FIC index. FIC index values were interpreted as follows: ≤0.5 indicated synergy, 0.5–1 indicated additive effects, 1–4 indicated no interaction (indifference), and >4 indicated antagonism.

### 2.7. Statistical Analysis

All statistical analyses were performed using R software (version 3.3.2; R Foundation for Statistical Computing, Vienna, Austria). Prior to hypothesis testing, data were screened for outliers, and the normality of residuals was assessed using the Shapiro–Wilk test, while homogeneity of variances was evaluated with Levene’s test. When assumptions of normality and homoscedasticity were met, differences among groups were analyzed using one-way analysis of variance (ANOVA). In cases where ANOVS indicated a significant main effect, Tukey’s HSD post hoc test was applied for pairwise comparisons. Statistical significance was set at *p* ≤ 0.05. Data are presented as mean ± standard deviation (SD). For clarity, results presented in the figures and text include specific significance levels (e.g., *p* < 0.05, *p* < 0.01, *p* < 0.001), and quantitative changes are expressed as percentages or fold changes relative to the control where appropriate.

## 3. Results

### 3.1. Assessment of Cell Viability

Treatment of primary canine PBMCs with different *E. coli* isolates (340, 404, and 863) did not lead to any significant increase in LDH release compared to the untreated control. Likewise, the flavonoid treatments—quercetin, luteolin, and proanthocyanidins tested at 25 and 50 µg/mL—did not elevate LDH levels relative to baseline. Since neither the bacterial treatments nor the flavonoids induced detectable cytotoxicity, all treatments were considered safe under the experimental conditions.

Treatment of primary canine PBMCs with different *E. coli* isolates (340, 404, and 863) did not result in significant changes in metabolic activity compared to the untreated control. The treatment with 0.5 µg/mL enrofloxacin did not affect cellular metabolic activity even after 24 h. The only exception was observed for the 404-isolate combined with enrofloxacin after 24 h of incubation, which resulted in a significant reduction in metabolic activity (*p* ≤ 0.05).

### 3.2. Effect of E. coli on Extracellular Hydrogen Peroxide and Intracellular ROS Production

**Figure 1:**
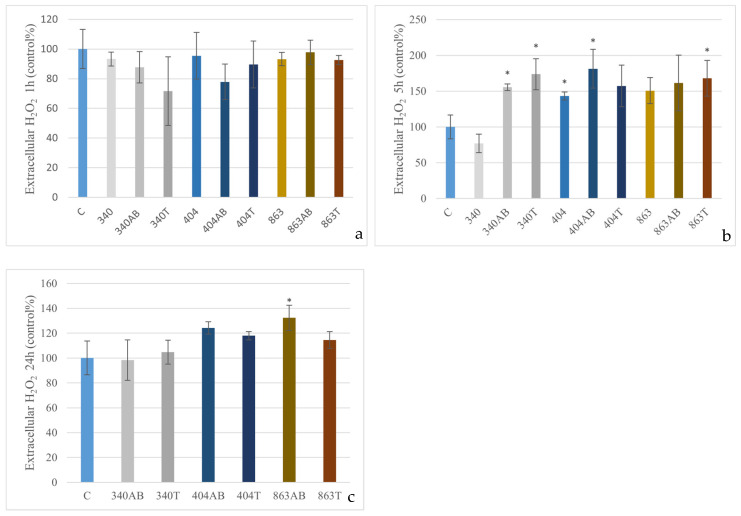
Effect of *E. coli* isolates (340, 404, 863) on extracellular H_2_O_2_ levels in canine PBMCs. Cells were exposed to live bacteria, enrofloxacin-treated (AB), or heat-inactivated (T) forms for 1 h (**a**), 5 h (**b**) and enrofloxacin-treated (AB), or heat-inactivated (T) forms for 24 h (**c**). Data are expressed as percentage of control (mean ± SD, *n* = 3). Significant differences from the control: * *p* < 0.05.

After 1 h of exposure, none of the treatments induced a significant change in extracellular H_2_O_2_ levels compared to the control. At 5 h, several groups showed a marked increase in H_2_O_2_ production: the enrofloxacin-treated and heat-inactivated forms of the 340 isolate, the untreated and enrofloxacin-treated variants of the 404 isolate, as well as the heat-treated 863 isolate all exhibited significantly elevated extracellular H_2_O_2_ levels (*p* < 0.05), with increases ranging from approximately 1.5-fold to nearly twofold relative to the control. After 24 h, the enrofloxacin-treated 863 isolate caused a significant increase in extracellular H_2_O_2_ compared to the control (*p* < 0.05), corresponding to roughly a 35% elevation (Figure 1).

**Figure 2:**
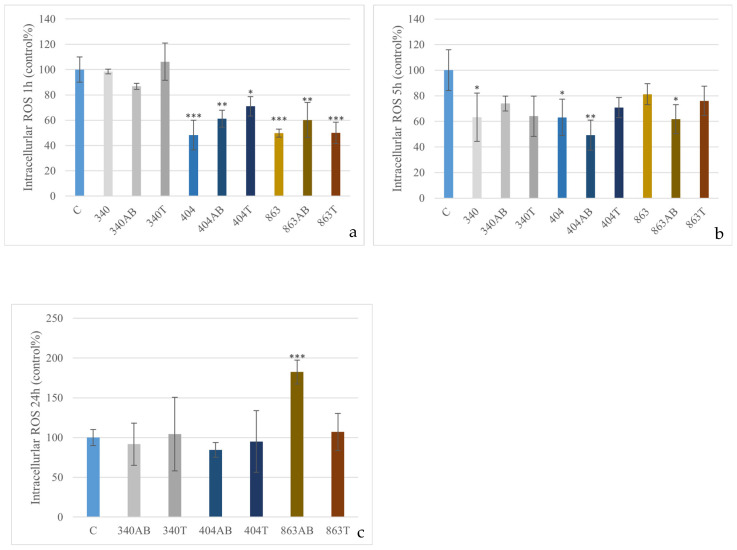
Effect of *E. coli* isolates (340, 404, 863) on intracellular ROS levels in canine PBMCs. Cells were exposed to live bacteria, enrofloxacin-treated (AB), or heat-inactivated (T) forms for 1 h (**a**), 5 h (**b**) and enrofloxacin-treated (AB), or heat-inactivated (T) forms for 24 h (**c**). Data are expressed as percentage of control (mean ± SD, *n* = 3). Significant differences from the control: * *p* < 0.05; ** *p* < 0.01; *** *p* < 0.001.

After 1 h of exposure, several treatments—particularly heat-inactivated bacteria and some antibiotic-treated groups—showed significantly lower ROS levels compared to the control (*p* < 0.05; *p* < 0.01; *p* < 0.001), with reductions of approximately 50%. At 5 h, a similar trend was observed, as some live, heat-treated, and antibiotic-treated groups again exhibited significantly decreased ROS generation (*p* < 0.05; *p* < 0.01), likewise reaching roughly half of the control levels. However, after 24 h, the enrofloxacin-treated 863 isolate significantly increased ROS production compared to the control (*p* < 0.001), resulting in nearly a twofold elevation (Figure 2).

### 3.3. Effect of E. coli on TNF-α Production

**Figure 3:**
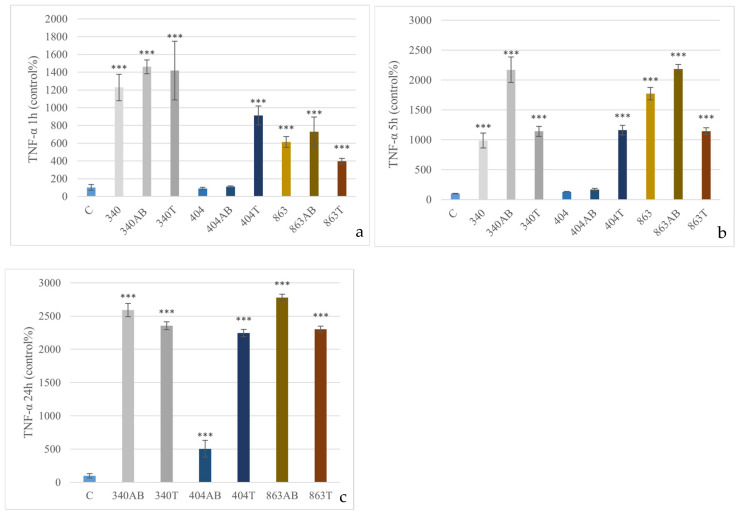
Effect of *E. coli* isolates (340, 404, 863) on TNF-α production in canine PBMCs. Cells were treated with live bacteria, enrofloxacin-treated (AB), or heat-inactivated (T) forms for 1 h (**a**), 5 h (**b**) and enrofloxacin-treated (AB), or heat-inactivated (T) forms for 24 h (**c**). TNF-α levels are expressed as percentage of the control (mean ± SD, *n* = 3). Significant differences from the control: *** *p* < 0.001.

The 340 and 863 isolates induced a significant increase in TNF-α concentration already after 1 h in all treatment forms (*p* < 0.001), corresponding to approximately a 12–14-fold and 4–6-fold rise, respectively. In contrast, for the 404 isolate, only the heat-inactivated form caused a significant elevation at 1 h, reaching roughly an eightfold increase (*p* < 0.001). At 5 h, all isolates and treatment forms triggered a marked rise in TNF-α levels—ranging from approximately 10- to 20-fold compared to the control (*p* < 0.001)—except for the untreated and antibiotic-treated forms of the 404 isolate, which did not differ significantly from the control. After 24 h, all isolates and treatment forms elicited a pronounced but comparatively lower increase in TNF-α levels, reaching about 20–25-fold above control values (*p* < 0.001), apart from the antibiotic-treated 404 isolate, which showed only 5-fold change (Figure 3).

### 3.4. Effect of Flavonoids on Extracellular Hydrogen Peroxide and Intracellular ROS Production

**Figure 4:**
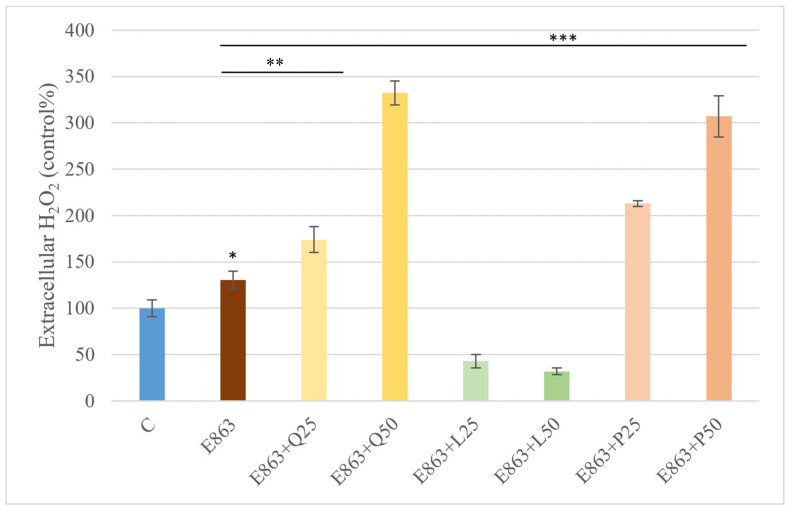
Effects of quercetin, luteolin, and proanthocyanidins on extracellular H_2_O_2_ levels in PBMCs stimulated with the *E. coli*. Data are presented as percentages relative to the control (mean ± SD, *n* = 3). E863: *E. coli* 863 isolate enrofloxacin-treated form. Q25, Q50: quercetin 25 and 50 µg/mL; L25, L50: luteolin 25 and 50 µg/mL; P25, P50: proanthocyanidins 25 and 50 µg/mL. Significant differences: * *p* < 0.05; ** *p* < 0.01; *** *p* < 0.001 compared to control group or *E. coli*-treated group.

Extracellular H_2_O_2_ production was assessed in PBMC cultures following stimulation with the enrofloxacin-treated *E. coli* 863 isolate. The bacterial treatment significantly increased extracellular H_2_O_2_ levels by approximately 35% compared to the control group (*p* < 0.05). Relative to the *E. coli*–treated group, quercetin further elevated H_2_O_2_ concentrations, with the 25 µg/mL dose inducing an additional 30% increase, while the 50 µg/mL concentration resulted in nearly a twofold elevation (*p* < 0.01). Proanthocyanidin treatment produced a similar pattern of enhancement at both tested concentrations. In contrast, luteolin markedly reduced extracellular H_2_O_2_ levels, lowering them to roughly one-quarter of those measured in the *E. coli*–treated group (*p* < 0.001) (Figure 4).

**Figure 5:**
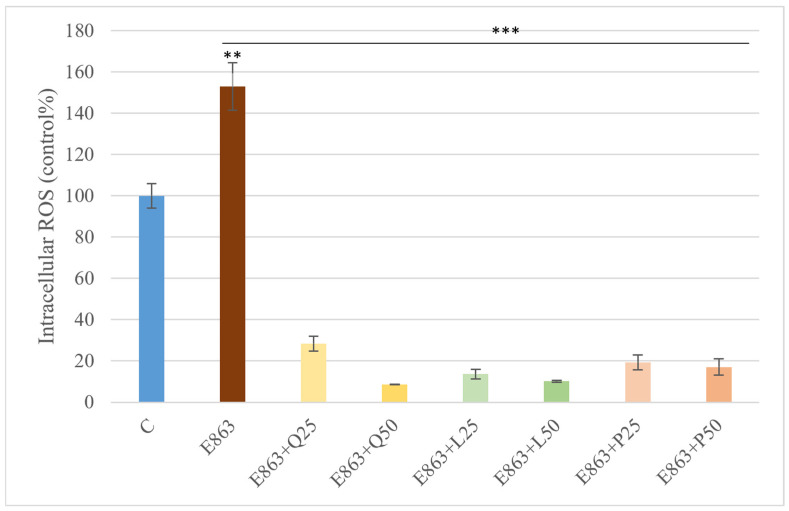
Effects of quercetin, luteolin, and proanthocyanidins on intracellular ROS levels in PBMCs stimulated with the *E. coli*. Data are presented as percentages relative to the control (mean ± SD, *n* = 3). E863: *E. coli* 863 isolate enrofloxacin-treated form. Q25, Q50: quercetin 25 and 50 µg/mL; L25, L50: luteolin 25 and 50 µg/mL; P25, P50: proanthocyanidins 25 and 50 µg/mL. Significant differences: ** *p* < 0.01; *** *p* < 0.001 compared to control group or *E. coli*-treated group.

Intracellular ROS generation in PBMCs was evaluated after stimulation with the enrofloxacin-treated *E. coli* 863 isolate. *E. coli* significantly increased ROS levels to approximately 1.5-fold compared to the control group (*p* < 0.01). Treatment with quercetin, luteolin, or proanthocyanidins at both tested concentrations (25 and 50 µg/mL) markedly reduced *E. coli*-induced ROS production to about one-sixth of the levels observed in the *E. coli*–treated group (*p* < 0.001) (Figure 5).

### 3.5. Effect of Flavonoids on TNF-α Production

**Figure 6:**
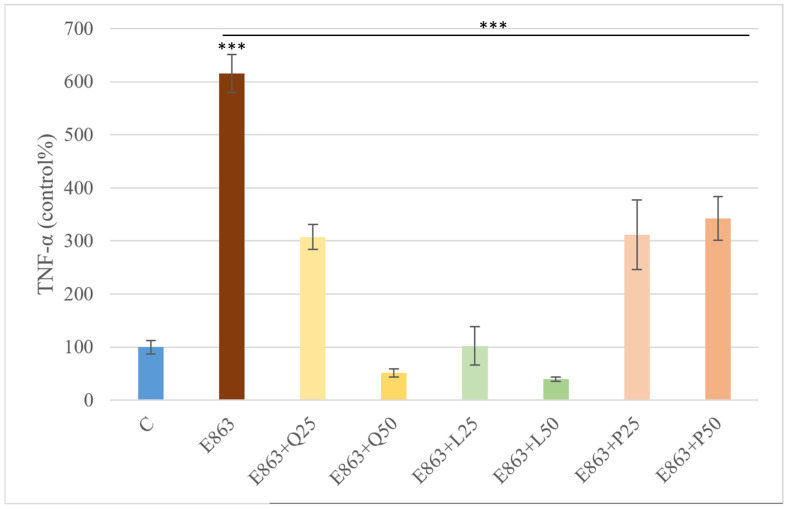
Effects of quercetin, luteolin, and proanthocyanidins on TNF-α levels in PBMCs stimulated with *E. coli*. Data are expressed as percentages relative to the control (mean ± SD, *n* = 3). E863: *E. coli* 863 isolate enrofloxacin-treated form. Q25, Q50: quercetin 25 and 50 µg/mL; L25, L50: luteolin 25 and 50 µg/mL; P25, P50: proanthocyanidins 25 and 50 µg/mL Significant differences: *** *p* < 0.001 compared to control group or *E. coli*-treated group.

Stimulation of PBMCs with the enrofloxacin-treated *E. coli* 863 isolate (0.5 µg/mL) resulted in a significant increase in TNF-α concentration, reaching approximately sixfold compared to the control (*p* < 0.001). All tested flavonoids—quercetin, luteolin, and proanthocyanidins—significantly suppressed *E. coli*-induced TNF-α production. Quercetin at 25 µg/mL and proanthocyanidin treatments reduced TNF-α levels to roughly half of the *E. coli*–treated group, whereas quercetin at 50 µg/mL and luteolin at both concentrations decreased TNF-α to less than one-quarter of the *E. coli*-induced levels (*p* < 0.001) (Figure 6).

### 3.6. Microbiological Analyses of the Investigated Flavonoids

The MIC and MPC values of quercetin, luteolin, and grape seed proanthocyanidins against the three canine-derived *E. coli* strains (340, 404, and 863) were investigated. Quercetin exhibited generally high MIC and MPC values (8192 µg/mL for isolates 340 and 863, and 2048 µg/mL for isolate 404), indicating limited antibacterial potential. Luteolin displayed moderate MICs (256 µg/mL for isolates 340 and 404) with markedly higher MPCs (2048 µg/mL and 4096 µg/mL, respectively), while both MIC and MPC values were high for isolate 863 (4096 µg/mL each). Grape seed proanthocyanidins showed lower MICs (512, 512, and 1024 µg/mL for isolates 340, 404, and 863, respectively), although their MPCs remained higher (1024, 512, and 1024 µg/mL). In contrast, enrofloxacin exhibited low MICs across all isolates (0.015625–0.03125 µg/mL), with MPCs of 0.03125 µg/mL for isolates 404 and 863, but a much higher MPC of 64 µg/mL for isolate 340. The combination assays revealed no interaction between enrofloxacin and any of the tested flavonoids, as FIC indices ranged between 1 and 2, indicating an indifferent effect.

## 4. Discussion

Chronic gastrointestinal inflammatory disorders in dogs require a multifaceted therapeutic approach that extends beyond symptom management to include preventive and microbiological strategies, encompassing the targeted control of oxidative stress, stabilization of the gut microbiome, and rationalized antibiotic use. The gut microbiome, comprising a diverse microbial community, is a key determinant of host health. Under physiological conditions, commensal bacteria suppress the overgrowth of pathogenic microorganisms while supporting enterocyte metabolism via short-chain fatty acid production. Although most strains are commensal and harmless, disruption of microbial eubiosis can lead to impaired gut barrier function and enable translocation of pathogenic species [7]. Such disruptions facilitate colonization of the mucosal surface by pathogenic microorganisms, including certain *E. coli* strains, triggering aberrant immune activation. Increased intestinal permeability allows pathogens and microbial components to enter the circulation, potentially causing invasive infections including septicemia [9]. *E. coli*-induced septicemia often arises secondary to GI dysregulation. During septic conditions, excessive ROS production and persistent inflammatory mediators induce oxidative stress, mitochondrial dysfunction, and cell death, collectively driving the progressive clinical manifestations observed in affected animals. Therapeutic intervention typically combines immunomodulators, anti-inflammatory drugs, systemic antioxidants, and, in urgent cases, empirical antibiotic therapy, which significantly reduces mortality. Targeted antibiotic therapy is feasible only after determining the antimicrobial susceptibility of strains isolated from hemocultures [36]. However, the time-intensive nature of susceptibility testing often necessitates first-line empiric treatments, with the growing prevalence of antibiotic resistance posing a significant challenge to treatment success. MDR strains represent a major global threat, with extended-spectrum β-lactamase-producing *E. coli* being particularly concerning [20].

Flavonoids are plant-derived compounds well recognized for their anti-inflammatory, antimicrobial, and immunomodulatory activities [37]. In our study, we investigated the antioxidant and anti-inflammatory effects of quercetin, luteolin, and grape seed proanthocyanidins using an in vitro canine white blood cell model. To simulate oxidative stress and inflammatory responses, we treated PBMCs with *E. coli* strains derived from canine septic infections (isolates 340, 404, and 863). The primary goal was to identify a bacterial strain that induces robust release of proinflammatory cytokines and ROS, suitable for subsequent flavonoid testing.

The impact of bacterial treatments on cellular metabolic activity and cytotoxicity was first assessed using LDH assay and CCK-8 kit, ensuring that no significant cytotoxicity was caused by any of the treatments. This is important because treatment-related changes in ROS under non-cytotoxic conditions are more likely to reflect regulated redox signaling and immune activation rather than nonspecific oxidative damage secondary to loss of viability. Following this, we quantified intracellular ROS levels in PBMCs exposed to *E. coli*. Notably, the only treatment eliciting a significant ROS increase relative to control was the 24 h exposure to isolate 863 combined with enrofloxacin, whereas shorter incubations often resulted in a reduction in ROS levels. One plausible explanation is a biphasic pattern—an early decrease followed by a late rise in ROS—can be interpreted as an initial adaptive response (often linked to Nrf2-associated cytoprotective programs and increased peroxide detoxification) that temporarily buffers oxidative signals, followed by a later pro-inflammatory amplification phase in which NF-κB/MAPK-driven activation and increased NOX/mitochondrial ROS production shift the redox balance toward higher ROS [38]. Similar time-dependent redox responses have been reported in immune cells exposed to microbial stimuli, where early antioxidant adaptation is followed by activation of oxidative signaling pathways [39]. This temporal shift likely reflects the transition from an adaptive to a pro-inflammatory phase of the host–pathogen interaction. The finding that only enrofloxacin-treated *E. coli* induced a significant intracellular ROS increase in PBMCs is consistent with evidence that bactericidal antibiotics generate substantial oxidative stress within bacteria. Fluoroquinolones disrupt DNA topology and central metabolism, leading to enhanced respiration, destabilization of iron–sulfur clusters, and ultimately hydroxyl radical formation—processes well documented across multiple species [40]. These stress pathways produce bacterial damage signals, including oxidized lipids, fragmented DNA, and other DAMP-like structures, which can be recognized by immune cells. Consequently, PBMCs exposed to antibiotic-stressed bacteria encounter a richer repertoire of immunostimulatory cues than those exposed to heat-killed bacteria, whose structural PAMPs are largely denatured or aggregated and therefore less effective in triggering ROS production. During the 24 h co-incubation, antibiotic-treated bacteria may continue to release stress-associated molecules, maintaining immune activation, whereas heat-killed bacteria remain inert. Moreover, some studies suggest that bactericidal antibiotics can also induce mitochondrial ROS in mammalian cells, potentially amplifying the host oxidative response [41]. However, when monitoring extracellular H_2_O_2_ levels, we observed a different pattern. After five hours, several treatments resulted in a significant increase, but after 24 h, this temporary increase subsided in most treatments, except for strain 863 AB, where an approximately 1.5-fold increase was observed. This strain also induced significant TNF-α elevation and was subsequently selected for further experiments. Because TNF-α is commonly regulated downstream of NF-κB and is also influenced by MAPK-dependent mechanisms, the concordant changes in ROS and TNF-α are compatible with engagement of redox-sensitive inflammatory networks [42]. PBMCs were then treated with quercetin, luteolin, and grape seed proanthocyanidins at concentrations of 25 and 50 µg/mL. All three flavonoids significantly reduced ROS and TNF-α levels at both concentrations compared to the *E. coli*-treated group. Extracellular H_2_O_2_ in PBMC supernatants showed a compound-specific pattern, increasing with quercetin and proanthocyanidins but decreasing with luteolin, despite a consistent reduction in intracellular ROS level at 24 h. This divergence is plausible because Amplex Red reports extracellular peroxide in an HRP-dependent reaction and polyphenols—particularly quercetin—can strongly interfere with HRP-coupled fluorescence assays, potentially leading to over- or underestimation of H_2_O_2_ depending on the compound and conditions. Luteolin’s effects may involve an enhanced antioxidant capacity through Nrf2-associated responses, as luteolin has been reported to promote Nrf2 nuclear translocation and increase the expression of Nrf2/ARE target genes such as HO-1 [43]. Overall, these results demonstrate treatment-associated modulation of oxidative and inflammatory outputs in bacterially challenged PBMCs, providing functional evidence for attenuation of the redox–inflammation axis under our experimental conditions. Comparable studies in human PBMC models stimulated with *E. coli* LPS have reported similar anti-inflammatory and antioxidant effects of quercetin and luteolin [36]. In murine models of rheumatoid arthritis, flavonoid treatments similarly decreased inflammatory factor secretion from immune cells [44]. Our results are consistent with previous canine PBMC evidence showing that flavonoids can attenuate LPS-induced oxidative and inflammatory responses. In an LPS-challenged canine PBMC model, quercetin and luteolin reduced TNF-α, and flavonoid co-treatment lowered intracellular ROS relative to LPS alone [45]. In our *E. coli*-challenged PBMC system, quercetin, luteolin, and grape seed proanthocyanidins similarly reduced ROS and TNF-α, extending these observations from endotoxin stimulation toward a bacterial trigger. Direct canine PBMC data with these specific flavonoids and endpoints remain limited; however, canine PBMCs are well established to mount robust pro-inflammatory cytokine responses (including TNF) to bacterial stimuli such as LPS or heat-killed bacteria, supporting TNF-α as a relevant readout in this species. Although we did not directly quantify pathway activation, NF-κB, MAPK, and Nrf2/ARE represent plausible mechanistic nodes linking the observed reductions in ROS and TNF-α. Dedicated pathway-focused analyses will be important next steps to delineate the relative contribution of these signaling routes in this PBMC model [46].

MIC and MPC determinations are essential for quantitatively assessing the antimicrobial potential of flavonoids. Our results suggest that luteolin exerts antibacterial activity at lower concentrations. Previous *E. coli* in vitro models reported quercetin as the most potent bacterial inhibitor, with luteolin showing weaker activity, likely due to structural differences [47]. However, antimicrobial effects are highly strain-dependent, and results may vary according to isolate origin. In vitro synergy testing between the flavonoids and enrofloxacin was also conducted, and a neutral interaction was observed. These findings suggest that co-administration with enrofloxacin is not contraindicated in experimental settings and may support future adjunctive therapy for septicemia.

These trends have prompted increasing interest in natural bioactive phytochemicals, which may offer alternative or adjunctive therapeutic options. Our data provide functional evidence that selected flavonoids can modulate the redox–inflammation axis in a PBMC model relevant to bacterial stimulation. Nevertheless, certain limitations should be considered when interpreting the present findings. The study relied on PBMCs isolated from a single healthy donor, and inter-individual variability in immune responsiveness may influence the reproducibility of cytokine and ROS profiles. Moreover, ex vivo conditions cannot fully replicate the complex interactions occurring in vivo, where gut microbiota composition, flavonoid metabolism, and host immune status jointly determine biological outcomes. In addition, the lack of dedicated pathway readouts limits mechanistic inference beyond the observed phenotype. Future studies should therefore validate these findings in vivo, determine effective dosing ranges, and link efficacy to systemic exposure by assessing pharmacokinetics and bioavailability; given variability in flavonoid absorption and metabolism, formulation and exposure–response analyses will be essential to evaluate translational potential. Despite these limitations, the canine PBMC model provides a valuable platform for probing immune redox and inflammatory modulation by flavonoids under bacterial challenge and for guiding subsequent in vivo investigations.

## 5. Conclusions

Overall, our results indicate that quercetin, luteolin, and grape seed-derived proanthocyanidins hold potential as adjunctive therapeutics in small animal medicine. Flavonoid interventions may be particularly relevant in GI disorders characterized by chronic inflammation, and risk of endotoxemia or septicemia. While in vitro models provide valuable mechanistic insights, conclusions should be interpreted cautiously considering methodological limitations. Our findings offer a foundational basis for designing more detailed investigations into flavonoid-based interventions. These findings highlight the relevance of the canine PBMC model for investigating immunomodulatory effects of flavonoids in bacterial inflammation.

## Data Availability

Data are contained within the article. Further inquiries can be directed to the corresponding author.
